# Comparison of Three Attractants for the Effective Capture of *Xylotrechus chinensis* Adults in Multi-Funnel Traps

**DOI:** 10.3390/insects14080676

**Published:** 2023-07-31

**Authors:** Nickolas G. Kavallieratos, Maria C. Boukouvala, Anna Skourti, Spyridon Antonatos, Panos V. Petrakis, Dimitrios P. Papachristos, Georgios Th. Papadoulis

**Affiliations:** 1 Laboratory of Agricultural Zoology and Entomology, Department of Crop Science, Agricultural University of Athens, 75 Iera Odos Str., 11855 Athens, Greece; annaskourti@aua.gr (A.S.); gpapadoulis@aua.gr (G.T.P.); 2 Laboratory of Agricultural Entomology, Department of Entomology and Agricultural Zoology, Benaki Phytopathological Institute, 8 Stefanou Delta Str., 14561 Kifissia, Greece; s.antonatos@bpi.gr (S.A.); d.papachristos@bpi.gr (D.P.P.); 3 Laboratory of Forest Entomology, Institute of Mediterranean Ecosystems, Hellenic Agricultural Organization—“Dimitra”, Terma Alkmanos, 11528 Athens, Greece; pvpetrakis@fria.gr

**Keywords:** blends, monitoring, tiger longicorn beetle, adult flight period, *Morus* spp.

## Abstract

**Simple Summary:**

Three blends of attractants and pheromones (i.e., attractant 1, attractant 2, and attractant 3) were compared for monitoring adults of *Xylotrechus chinensis*. The experimental procedure was carried out for a two-year period. The attractants were baited in traps and placed in trees of three heavily infested areas of Athens (Greece). Traps were checked weekly, the captured *X. chinensis* adults were recorded, and they were removed from the traps. Adults of *X. chinensis* start flying in late spring and end in the middle of autumn (end of October). Attractant 3 performed better than attractant 1 and 2, even with low numbers of insects. *Xylotrechus chinensis* did not seem likely to be attracted to the basic part of the blend, which consisted of a blend of pheromones + ethanol. Overall, the inclusion of α-pinene and ipsenol increased the activity of the basic lure.

**Abstract:**

The Asian coleopteran *Xylotrechus chinensis* (Chevrolat) (Cerambycidae: Cerambycinae) is an invasive species in several European countries, attacking mulberry trees. In the current research, we evaluated the performance of three mixtures consisting of pheromones and attractants for the monitoring of *X. chinensis* adults. Attractant 1 (i.e., geranyl acetone, fuscumol acetate, fuscumol, monochamol, 3-hydroxyhexan-2-one, 2-methyl-1-butanol, *anti*-2,3-hexanediol, prionic acid + ethanol), attractant 2 (i.e., geranyl acetone, fuscumol acetate, fuscumol, monochamol, 3-hydroxyhexan-2-one, 2-methyl-1-butanol, *anti*-2,3-hexanediol, prionic acid + α-pinene + ethanol) and attractant 3 (i.e., geranyl acetone, fuscumol acetate, fuscumol, monochamol, 3-hydroxyhexan-2-one, 2-methyl-1-butanol, *anti*-2,3-hexanediol, prionic acid + α-pinene + ipsenol + ethanol) were baited in multi-funnel traps and installed in mulberries for a two-year period in Athens (Greece). The flight activity of *X. chinensis* starts at the end of April and terminates at the end of October. The peaks of *X. chinensis* flight activity were observed on 16 August 2021 and on 6 July 2022. Attractant 3 proved to be the most effective blend, catching 953 adults, followed by attractant 2 (523 adults) and attractant 1 (169 adults), throughout the experimental period. It seems that the pest was not attracted to the basic part of the blend (i.e., pheromones + ethanol). The incorporation of α-pinene and ipsenol resulted in the elevated activity of the base lure. The elevated performance of attractant 3 may be attributed to only the α-pinene and the ipsenol, or possibly the α-pinene, ipsenol, and ethanol, because the pheromone blend did not contain any of the pheromone components of the target species. Overall, attractant 3 could be a useful tool to detect and track *X. chinensis* in new invasive areas, triggering early management strategies against further establishment of this species.

## 1. Introduction

The invasive species *Xylotrechus chinensis* (Chevrolat) (Coleoptera: Cerambycidae: Cerambycinae) is a destructive pest that feeds and grows on mulberries (*Morus* spp.) [1,2,3]. This species is native to the Eastern Palaearctic region (i.e., China, South Korea, and Japan) [4,5,6]. In the last decade, *X. chinensis* has become established in Europe, mainly in countries with a wide presence of *Morus* spp., such as France, Greece, and Spain [1,2,7,8,9,10]. Specifically, in Spain, *X. chinensis* was first recorded in 2013 in Catalonia, where in 2020 the infestation had expanded to 12 cities, covering an area of 378.1 km^2^ [11]. Meanwhile, a new outbreak of *X. chinensis* occurred in Valencia in 2018 [2]. In the same year, *X. chinensis* was reported for the first time in France (Gironde) [12,13], while in 2019 attacked *Morus* trees were found in the Occitanie region [8,14]. The first detection of this species in Greece was in 2017 in Crete, where approximately 200 mulberries (*Morus* spp.) were found infested, of which 15% were dead [1]. In 2019, an outbreak of *X. chinensis* was recorded in Athens, the capital of Greece, where 300 out of 1300 infested mulberry trees were dead [9,13]. The damage from *X. chinensis* spread further in Athens in the following years, making this species an important pest of mulberry trees throughout the city. In addition, *X. chinensis* has been intercepted twice in Germany, in 2007 (first record in Europe) and 2017, and once in Philadelphia, USA (2011) in containers transporting different wooden objects from China [6,11,15], indicating that wooden materials can be vehicles for its spread.

Early detection of *X. chinensis* is a complex issue, since the immature stages develop inside the tree trunk [8]. The infestation is usually noticed mainly by the exit holes of the adults, as well as by other symptoms that are not so obvious, such as frass and the droppings of the beetles on the trunk [1,2,10]. The xylophagous larvae inhibit the movement of nutrients and water within the infested trees, resulting in their progressive drying [16]. The feeding habit of *X. chinensis* adults is not clear, since it is reported that adults infest leaves and stems of the tops of the mulberry trees [1,2] or they do not feed but drink water [17,18], indicating that the most destructive life stage of this species is larva. For all the above reasons, management of *X. chinensis* is challenging. In recent research efforts in Spain (Barcelona) and Greece (Athens), the trunk injection method was used to control *X. chinensis* larvae [10,11]. During spring 2018 in Barcelona, abamectin was injected into 107 mulberry trees. Almost a third of these treated trees (31 out of 107) showed new infestation symptoms in December, particularly exit holes, indicating a significant reduction in *X. chinensis* presence [11]. In Athens, after two years of application of abamectin, fipronil and imidacloprid via trunk injection to infested *Morus* spp. trees, the emergence holes of *X. chinensis* adults were reduced by 85.6%, 71.8%, and 76.1%, respectively [10]. Chemical control of this species has previously been proposed by spraying the trunks of mulberry trees with contact insecticides, targeting sites where females are resting and/or laying eggs, and newly emerged larvae are present [2]. Both methods were applied during summer [2,10,11]. The trunk injection method is generally safe and can be applied to urban trees [10,19], while trunk spraying is not suggested in urban areas due to concerns related to the exposure of citizens and environment to insecticides [20]. In addition, some preventive measures should be applied to heavily infested trees, including cutting down the affected mulberries, followed by burning or shredding the removed parts of the trees [2,8,12].

Recently, a new device that can monitor trees systematically against wood-boring pests was evaluated [21]. Trees infested by *X. chinensis* and *Rhynchophorus ferrugineus* Olivier (Coleoptera: Curculionidae) were used in the tests. This device automatically records the acoustic emissions generated by the activity of the larvae while feeding inside the trunk of the tree, thus detecting any tree infestation. It showed high accuracy in the detection of wood borer species and provided rapid inspection of many mulberries and palms without human interference, rendering it particularly suitable for trunk inspection in quarantine areas. The vibration recording device potentially contributes to a reduction in unnecessary management treatments and becoming a useful tool for monitoring of *X. chinensis*.

The three ingredients of male pheromones of *X. chinensis,* i.e., 2,3-octanediol, 2-hydroxy-3-octanone, and 3-hydroxy-2-octanone [22,23], have been identified and attract female individuals [24], but they have not yet been developed as a method of detection for this species [8]. Pheromone components could be used for mass trapping of *X. chinensis* females or for mating disruption [8]. Recently, traps containing a mixture of eight kairomones and pheromones, which attracted a wide spectrum of insects [25], were used in Catalonia (Spain), Crete (Greece), and Sète (France) [8,14]. Among the captured species of the Cerambycidae family were *X. chinensis* adult individuals.

In the present study, three mixtures of attractants that can attract a wide range of wood-boring insects were compared to monitor the population of *X. chinensis* in different areas of the Municipality of Athens. It is very important to know whether any of the components that make up each attractant can disrupt the attraction of *X. chinensis* [26]. A search of the global bibliography yielded no data on the seasonal occurrence of *X. chinensis* in Europe. There are sporadic reports on the emergence period of *X. chinensis* adults from the trunks of the mulberries, providing evidence about the beginning of the flight of this pest. In southern Greece, adults of *X. chinensis* appear from the end of spring to the beginning of summer and in northeastern Spain from mid- to late summer [1,2]. Thus, the aim of the current two year (2021–2022) research was to investigate the flight activity of *X. chinensis*, offering a useful monitoring tool for this species.

## 2. Materials and Methods

### 2.1. Experimental Areas-Trees

**Figure 1:**
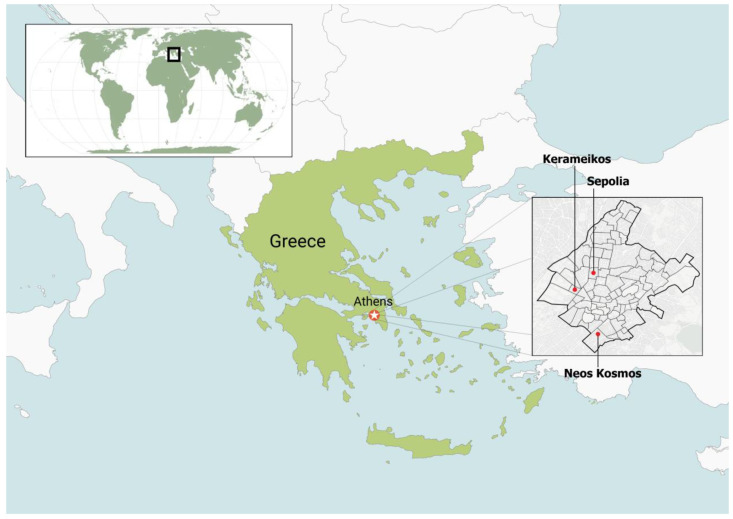
Map of Greece with the three experimental areas: Neos Kosmos, Kerameikos, and Sepolia, in the Municipality of Athens.

Three experimental areas were selected within the Municipality of Athens: Neos Kosmos (37° 03′ 60.00″ N 22° 25′ 59.99″ E), Kerameikos (37° 58′ 25.19″ N 23° 43′ 4.19″ E), and Sepolia (38° 00′ 9.99″ N 23° 42′ 48.58″ E) (Figure 1). The three selected areas have *Morus* spp. trees (*Morus alba* and *Morus nigra*). Based on the existence of numerous exit holes in mulberry trees, preliminary samplings (through installation of baited traps) indicated that mulberry trees suffered high infestation levels of *X. chinensis* in these areas.

### 2.2. Attractants

The following three blends of pheromones and attractants were used during the experimental period: (i) geranyl acetone, fuscumol acetate, fuscumol, monochamol, 3-hydroxyhexan-2-one, 2-methyl-1-butanol, *anti*-2,3-hexanediol, prionic acid + ethanol (attractant 1); (ii) geranyl acetone, fuscumol acetate, fuscumol, monochamol, 3-hydroxyhexan-2-one, 2-methyl-1-butanol, *anti*-2,3-hexanediol, prionic acid + α-pinene + ethanol (attractant 2); and (iii) geranyl acetone, fuscumol acetate, fuscumol, monochamol, 3-hydroxyhexan-2-one, 2-methyl-1-butanol, *anti*-2,3-hexanediol, prionic acid + α-pinene + ipsenol (2-methyl-6-methylene-7-octen-4-ol) + ethanol (attractant 3). The three attractants had components in common of geranyl acetone, fuscumol acetate, fuscumol and monochamol, which are components of the pheromones of various species of the cerambycid subfamilies Spondylidinae and Lamiinae [27,28,29], 3-hydroxyhexan-2-one, 2-methyl-1-butanol, and *anti*-2,3-hexanediol, which are components of the pheromones of various species of the subfamily Cerambycinae [30,31,32], prionic acid, which is the sex pheromone of various species of the genus *Prionus* (Coleoptera: Cerambycidae: Prioninae) [33], and ethanol, which is released from infested trees [34,35,36], enhancing the attraction of wood-boring insects [37,38]. Attractant 3 additionally contained the components α-pinene, which is the kairomonic component released by host trees [39] and ipsenol, which is an aggregation pheromone of wood-boring insects of the subfamily Scolytinae (Curculionidae) [40,41]. Attractant 2 contained only α-pinene as an additional component. We assume that the blend of pheromones and ethanol was enhanced by α-pinene and ipsenol [42,43,44].

**Figure 2:**
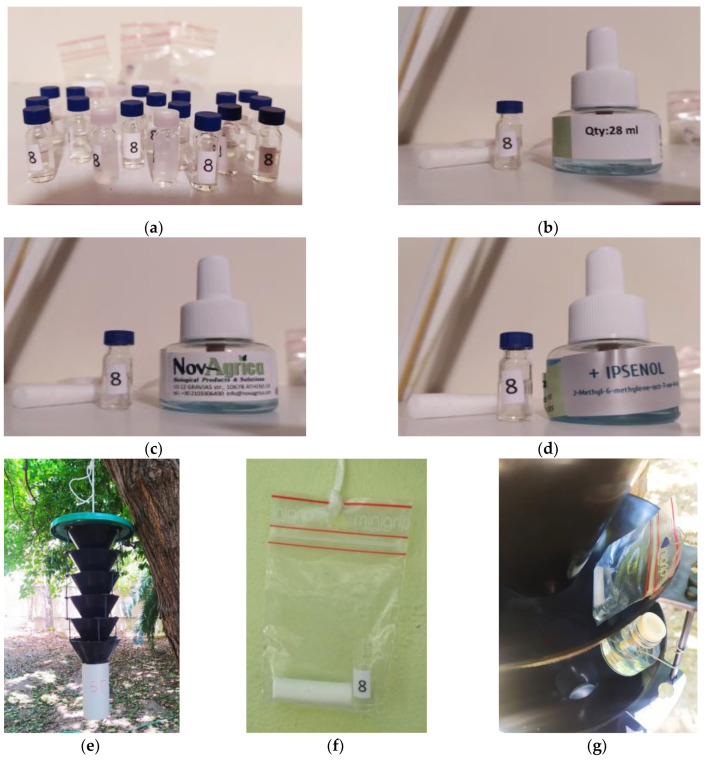
(**a**) The common part of the three attractants in glass vials. (**b**) Attractant 1. (**c**) Attractant 2. (**d**) Attractant 3. (**e**) Multi-funnel trap. (**f**) The common part of the three attractants in a glass vial in a polyethylene bag with cotton wad. (**g**) Placement of attractant in the middle funnel of the multi-trap.

The preparation of the common part of the three attractants was carried out at the French National Institute of Agriculture, Food and the Environment (INRAE) by Dr Alain Roques. This blend consisted of 25 mg of geranyl acetone, 50 mg of fuscumol acetate, 50 mg of fuscumol, 50 mg of monochamol, 50 mg of 3-hydroxyhexan-2-one, 50 mg of 2-methyl-1-butanol, 50 mg of *anti*-2,3-hexanediol, and 1 mg of prionic acid dissolved in isopropanol (carrier) to a final volume of 1 mL. Aliquots of 1 mL of this mixture were poured into 1.5 mL glass vials with plastic lids and kept at 4 °C until the beginning of the experiment (Figure 2a). The extra components α-pinene (12 mL) (+ 16 mL ethanol) of attractant 2 or α-pinene (12 mL) and ipsenol (20 mg) (+ 16 mL ethanol) of attractant 3 are commercially available and contained in vials of 28 mL volume each with a constant evaporation rate of average 150 mg/day, lasting for 100 days, as measured by the manufacturer (NovAgrica, Athens, Greece). Ethanol alone as part of the first attractant is contained in the same type of vials (28 mL volume). The vials with ethanol alone were inspected frequently and filled if it was necessary. Therefore, per attractant two vials were used: attractant 1, a 1.5 mL vial containing the common part and a 28 mL vial containing ethanol (Figure 2b); attractant 2, a 1.5 mL vial containing the common part and a 28 mL vial containing α-pinene + ethanol (Figure 2c); and attractant 3, a 1.5 mL vial containing the common part and a 28 mL vial containing α-pinene + ipsenol + ethanol (Figure 2d). Upon installation of traps, the caps of the 28 mL vials were removed and the vials left open.

### 2.3. Experimental Design

The commercially available multi-funnel traps, which are suitable for catching species of cerambycids [45], were supplied by NovAgrica (Athens, Greece) and were used in the experiment. The multi-funnel trap consists of six black funnels with a removable white collection cup that facilitates the counting of the captures (dry trapping) (Figure 2e). A killing agent (transfluthrin 0.4% *w*/*w* strip) was applied in the collection cup to prevent captured insects from escaping. The traps were hung from trees with their bottoms 2.5 m above the ground. In the areas of Neos Kosmos and Kerameikos, 6 traps were placed for each attractant (i.e., 6 traps × 3 attractants × 2 areas = 36 traps), while in Sepolia, 3 traps were placed for each attractant (i.e., 3 traps × 3 attractants x 1 area = 9 traps). In each area, traps were suspended on main roads in blocks consisting of the three attractants and were 100 m apart (i.e., 6 blocks for Neos Kosmos and Kerameikos, 3 blocks in Sepolia). The distance between the blocks was 100 m. Before the suspension of the traps on mulberry trees, each vial of 1.5 mL containing the common part of the attractants was placed without the lid in a 65 mm × 95 mm, 100 µm thickness, resealable polyethylene bag (Minigrip bags, Avantor, The Metropolis, Singapore) with a cylindrical cotton wad (3.5 cm × 1 cm) [46] (Figure 2f). Attractants were placed in the middle funnel (Figure 2g) of the trap and replaced every three months. Trap inspections were conducted weekly from 19 July 2021 to 31 December 2022. At each trap check, traps were clockwise rotated to avoid the effect of a particular sampling location [47]. At each check, captures of *X. chinensis* adults were recorded. Then, all captured individuals were removed from the traps.

### 2.4. Statistical Analysis

Before the data of captures were analyzed, they were log (x + 1)-transformed to normalize variances and standardize means [10,48]. Data were analyzed using a three-way ANOVA [49]. The response variable was the number of captured adults. Main effects were attractant, date and experimental area. Their interactions (area x attractant, area x date, attractant × date, and area × attractant × date) were also considered in the analysis. Year was not considered in the analysis, since 2021 included fewer checks than 2022. Means were separated by the Tukey–Kramer HSD test, *p* = 0.05 [50]. All dates with zero captures in any treatment were dropped from the analysis. The analyses were carried out with the statistical package JMP 16.2 [51].

## 3. Results

**Figure 3:**
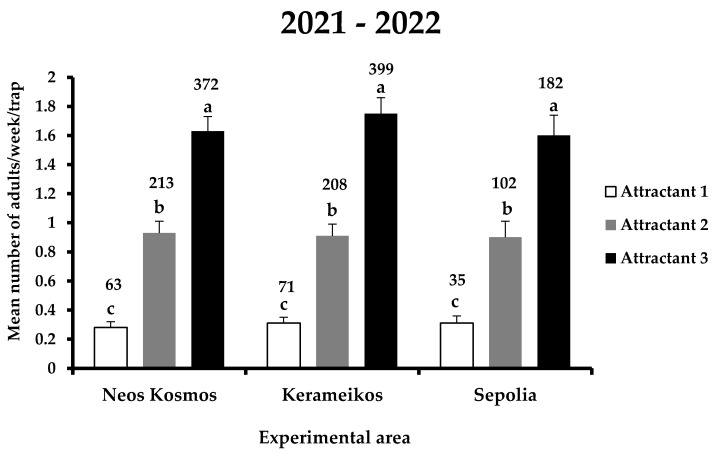
Mean number (+ standard error) of *X. chinensis* adults captured weekly in traps baited with each attractant in the three experimental areas (i.e., Neos Kosmos, Kerameikos, and Sepolia) during the entire experimental period (2021–2022). Different letters indicate significant differences. Numbers above columns indicate the total number of captures. All dates when all treatments had zero catches were dropped.

The main effects area and the interactions area x attractant and area x attractant x date were not significant (Table 1). The total number of *X. chinensis* adults captured in the traps for the entire experimental period was 1645, of which 169, 523, and 953 individuals were recorded in attractants 1, 2, and 3, respectively (Figure 3). Within each experimental area, significant differences were observed among the three attractants (*F* = 51.5; DF = 8, 1709; *p* < 0.01) (Figure 3). In Neos Kosmos, the most effective was attractant 3, catching a total of 372 adults, followed by attractant 2 and 1 with 213 and 63 individuals captured, respectively. The same trend was observed in Kerameikos and Sepolia, where the most catches were noted for attractant 3 (399 and 182 adults in Kerameikos and Sepolia, respectively) and the least for attractant 1 (71 and 35 adults in Kerameikos and Sepolia, respectively). No significant differences were recorded for each attractant among the three experimental areas, indicating that there was no influence of area on the performance of attractants.

**Figure 4:**
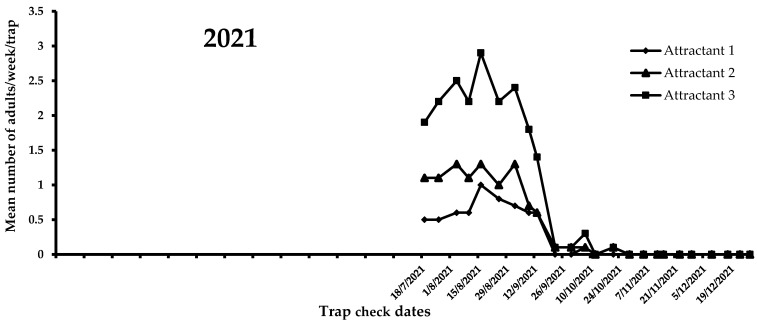
Mean number of *X. chinensis* adults captured weekly in traps baited with attractants 1, 2, and 3 during 2021.

**Figure 5:**
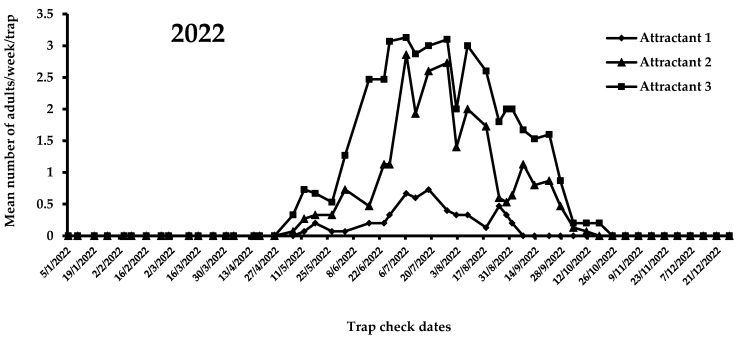
Mean number of *X. chinensis* adults captured weekly in traps baited with attractants 1, 2, and 3 during 2022.

A view of the flight activity of *X. chinensis* recorded in 2021 and 2022 in Athens is shown in Figure 4 and Figure 5. The peaks of captures for 2021 and 2022 were observed in August and July, respectively. Specifically, the mean numbers of captures for August 2021 were 2.47, 1.15, and 0.75 for attractants 3, 2, and 1, respectively. In July 2022, the mean numbers of captures were 3.03, 2.53, and 0.60 for attractants 3, 2, and 1, respectively. During 2021, significant differences were noted among captures in traps baited with the three tested attractants (*F* = 51.6; DF = 2, 584; *p* < 0.01). Traps baited with attractant 3 (i.e., 303 adults) caught significantly more adults than traps baited with attractant 1 (i.e., 89 adults) and 2 (i.e., 148 adults) (Figure 4). The highest mean number of *X. chinensis* adults captured from the beginning of observations until 13 September 2021 was recorded for attractant 3, followed by captures corresponding to attractant 2 and attractant 1 (Figure 4). The maximum mean number of catches was recorded on 16 August 2021, where 2.9 adults/week/trap were captured in traps baited with attractant 3. From 22 September 2021 onwards, a reduction in captures of *X. chinensis* was observed for all three tested attractants. From 29 October 2021 until 26 April 2022, there were zero captures (Figure 4 and Figure 5). A similar trend was recorded in 2022. There were significant differences in captures when traps were baited with attractants 1, 2, and 3 (*F* = 160.6; DF = 2, 1124; *p* < 0.01). Traps containing attractant 3 caught significantly more adults vs. traps containing attractant 2 vs. traps containing attractant 1 (Figure 5). The highest mean number of adults captured from the beginning to the end of *X. chinensis* flight activity was recorded for attractant 3, followed by captures corresponding to attractant 2 and attractant 1 (Figure 5). The first captures for attractants 2 and 3 occurred on 6 May 2022 (0.33 and 0.07 adults/week/trap for attractants 3 and 2, respectively), while traps baited with attractant 1 caught the first adults (0.07 adults/week/trap) one week later. The highest mean numbers of captured *X. chinensis* adults were noted on 6 July 2022 for attractants 2 and 3 (2.86 and 3.13 adults/week/trap for attractants 2 and 3, respectively), while for attractant 1 this was observed on 18 July (0.73 adults/week/trap). The last catches were observed on September 1, 2022 for attractant 1 (0.2 adults/week/trap), 11 October for attractant 2 (0.07 adults/week/trap), and 18 October for attractant 3 (0.2 adults/week/trap). No captures were recorded from 25 October to the end of December 2022.

**Figure 6:**
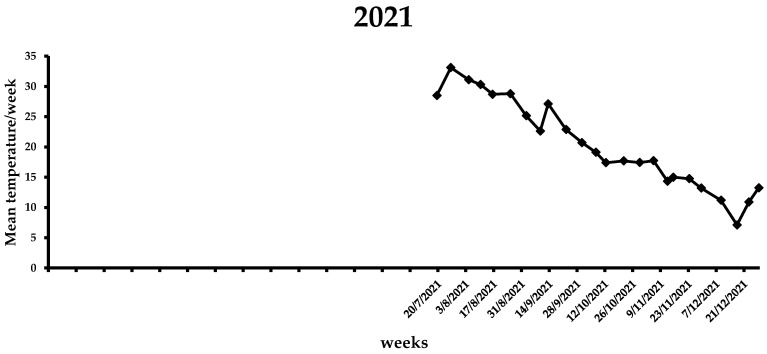
Mean weekly temperature (°C) in Municipality of Athens during the 2021 experimental period. Data obtained from freemeteo.gr.

**Figure 7:**
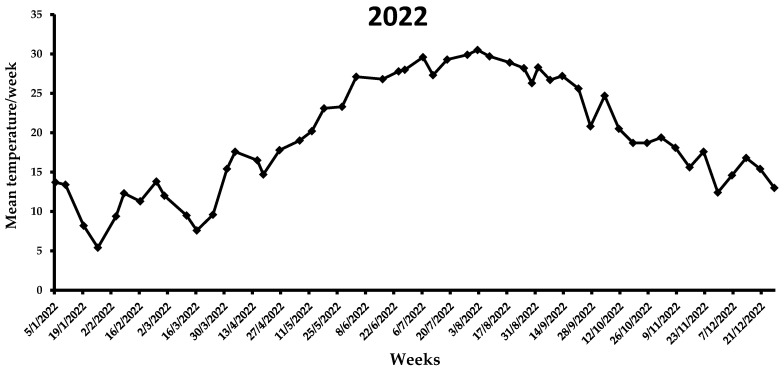
Mean weekly temperature (°C) in Municipality of Athens during the 2022 experimental period. Data obtained from AccuWeather.com.

During the first week of the observations in 2021 (i.e., end of July), the mean weekly temperature was 28.5 °C (Figure 6), which increased in the following three weeks, ranging from 30.3 to 33.1 °C. A gradual reduction in the average weekly temperature was noted from middle August (i.e., 28.7 °C) to the end of 2021 (i.e., 7.1 °C). By the end of the flight period in 2021, the mean weekly temperature was <20 °C. In the first two weeks of 2022, the average weekly temperature increased, reaching ~13 °C, while for the following three weeks it remained <10 °C (Figure 7). The same trend was observed over the next 7 weeks, ranging from 7.6 to 13.8 °C. A gradual increase in the mean weekly temperature was observed between the end of March till 26 April, reaching 17.8 °C. During the first two weeks of May, where the first captures were recorded, the mean weekly temperature increased further, reaching 20.2 °C. In the following weeks until the beginning of August, the mean weekly temperature ranged from 23.1 to 30.5 °C, while it gradually decreased after the second week of August. Similarly to 2021, the mean weekly temperature dropped below 20 °C by the end of the flight period of *X. chinensis*.

## 4. Discussion

The findings of the current study revealed that the most effective attractant mixture was attractant 3, which contained the two additional components α-pinene and ipsenol (+ethanol). When ipsenol was absent (attractant 2) or when α-pinene and ipsenol were absent (attractant 1), captures were significantly fewer than those corresponding to attractant 2. Captures corresponding to attractant 2, which contained α-pinene (+ethanol), were significantly higher than the captures corresponding to attractant 1 (+ethanol). Therefore, the combination of α-pinene and ipsenol with the common part of all attractants and ethanol resulted in the increased attraction of *X. chinensis* adults compared with the presence of α-pinene with the common part of the attractants + ethanol. Ethanol and α-pinene perhaps act synergistically to attract many species of wood-boring and bark beetles, including species of the family Cerambycidae, issues that have classified them as broad-spectrum combinational attractants [43,52,53]. According to Rodríguez-González et al. [42], the addition of ethanol to the compound 3-hydroxy-2-hexanone, which is produced by the males of *Xylotrechus arvicola* (Olivier) (Coleoptera: Cerambycidae: Cerambycinae), increased the attractiveness of this compound to both sexes of this species. In previous studies, it has been noted that the kairomones ethanol and α-pinene increased the attractiveness of monochamol, which is a component of *Monochamus galloprovincialis* Olivier (Coleoptera: Cerambycidae: Lamiinae) pheromone [54,55]. Hoch et al. [46] reported that the inclusion of α-pinene and ethanol in a mixture of geranyl acetone, fuscumol acetate, fuscumol, monochamol, 3-hydroxyhexan-2-one, 2-methyl-1-butanol, *anti*-2,3-hexanediol, and prionic acid significantly increased the number of captured individuals of species of the family Cerambycidae compared to the captures recorded in the mixture without the addition of kairomones. In our study, the addition of ipsenol to attractant 3 further enhanced its effectiveness. Similarly, Miller et al. [44] reported that the number of captured individuals of *Monochamus scutellatus* (Say) (Coleoptera: Cerambycidae: Lamiinae) in traps that had a mixture of ipsenol and α-pinene was higher than the number of captures in traps that had α-pinene alone. The authors also found that ipsenol significantly increased the number of captures of *Acanthocinus princeps* (Walker), *Acanthocinus obliquus* (LeConte), *Acanthocinus nodosus* (F.), *Acanthocinus obsoletus* (Olivier) (Coleoptera: Cerambycidae: Lamiinae), and *Rhagium inquisitor* (L.) (Coleoptera: Cerambycidae: Lepturinae) when α-pinene was added to the traps.

One other important finding of the present study was the depiction of the flight activity of *X. chinensis*, revealing valuable data on the ecology of this pest that could be useful for its successful management. Specifically, during the monitoring of the traps, it was found that the flight of *X. chinensis* begins around the end of April and terminates around the end of October. This stands in agreement with the suggestion of Leivadara et al. [1] for the emergence of *X. chinensis* in Crete (Greece), which takes place between May and June. In Spain (Catalonia), Sarto i Monteys and Torras i Tutusaus [2], after two years of observations of specimens kept in cages under semi-field environment conditions, reported that adults emerge later, in July and August, exactly the same period as adults of *X. chinensis* emerge in Japan, which is one of the countries of origin of this species [22]. The authors also found that in both years of their observations, the emergence of adults began after ten or seven consecutive days with mean daily temperature >20 °C, for 2016 and 2017, respectively [2]. In Athens, the first captures of traps were recorded at the beginning of May 2022. In the time between the previous check (late April) of the traps and the check of the first captures, it was observed that the average daily temperature in Athens was ~20 °C. New adults of *X. chinensis* after eclosion have been reported to remain inside the trees for a period between 12 and 40 days before leaving the trees [17,18], probably awaiting stabilization of the temperature above 20 °C. Hence, considering our results and those obtained from Spain, we assume that emergence of *X. chinensis* adults from infested trees requires certain temperature conditions. Additional research is required to verify this hypothesis.

Concerning the termination of the flight period of *X. chinensis*, in both years of the study, the last captures were recorded between 18 and 21 October. Although the life span of *X. chinensis* adults is not known, considering the period of adults’ emergence and the flight activity of *X. chinensis* obtained in our experiments, we assume that the last adults that emerged from the trees correspond to the last individuals captured in the traps. This issue should be further investigated. Regarding the performance of each attractant, attractant 1 ceased to be active in early September. The early reduction in the captures of attractant 1 compared to attractants 2 and 3 is probably due to the low performance exhibited since the beginning of *X. chinensis* flight activity. It should be noted that the last captures in 2022 were recorded in traps containing attractant 3, although in 2021 the last captures were recorded in traps containing either attractant 2 or attractant 3. Having expanded 334 km^2^ in Catalonia in two years, *X. chinensis* is expected to spread rapidly to nearby areas [8,11,55] and through wooden boxes that are used for the transportation of goods [16]. Therefore, attractant 3 could be used to detect early arrivals of the pest. Precise records of *X. chinensis* in new areas could trigger management measurements to restrict further dispersal of this species.

## 5. Conclusions

To conclude, the most effective attractant was found to be attractant 3, which can help the successful monitoring of *X. chinensis*. The combination of α-pinene and ipsenol enhanced the activity of the basic part of the blend (i.e., pheromones + ethanol), while the role of ethanol is rather uncertain. It does not seem likely that *X. chinensis* was attracted to the basic part of the blend, because it was the least active attractant. Thus, it seems more likely that the pest was attracted to the blend of kairomones, with the data showing that α-pinene and ipsenol were attractive. Further investigation is needed by testing other blends of attractants. In addition, the role of α-pinene, ipsenol, and ethanol should be evaluated for the attraction of *X. chinensis* to make a direct comparison to attractant 3. The precise monitoring of populations of noxious insect populations is crucial, since it provides timely and detailed information on pest densities, resulting in a reduction in synthetic insecticides and the prompt application of ecologically friendly methods, such as mass trapping. The next step of investigation deals with the assessment of new trap devices to optimize the attractant-based trapping protocol of this noxious species.

## Figures and Tables

**Table 1:** ANOVA parameters for main effects and their interactions for *X. chinensis* adults captured weekly in each trap for attractants in three experimental areas during 2021–2022 (total DF = 1709).

Source	DF	*F*	*p*
Area	2	0.2	0.84
Attractant	2	294.2	<0.01
Date	37	20.6	<0.01
Area × attractant	4	0.4	0.83
Area × date	74	1.6	0.01
Attractant × date	74	3.3	<0.01
Area × attractant × date	148	1.0	0.54

## Data Availability

Data are contained within the article.
